# A COVID-19 Survey among People Who Use Drugs in Norway

**DOI:** 10.3390/ijerph19127002

**Published:** 2022-06-08

**Authors:** Gabrielle K. Welle-Strand, Linn Gjersing, Ida K. Olsen, Thomas Clausen

**Affiliations:** 1 Norwegian Centre for Addiction Research (Seraf), Postboks 1039 Blindern, 0315 Oslo, Norway; thomas.clausen@medisin.uio.no; 2 Norwegian Institute of Public Health, 0213 Oslo, Norway; linn.gjersing@fhi.no; 3 ProLAR Nett, 4640 Søgne, Norway; ida@prolar.no

**Keywords:** people who use drugs, survey, COVID-19 recommendations, testing and vaccination, Norway

## Abstract

Background: to combat the COVID-19 pandemic, adherence to COVID-19 recommendations and vaccination against COVID-19 have been important. Among people who use drugs (PWUD), little is known regarding compliance towards COVID-19 recommendations, COVID-19 testing, or attitudes towards COVID-19 vaccination. We aimed to examine these issues in a sample of PWUD. Methods: a cross-sectional study was conducted between January and March 2021. Through users’ organizations and different low-threshold services for substance users, we recruited former drug users and professionals to include participants and perform the interviews. Participants completed an interviewer-administered questionnaire. Any person above 16 years of age who used substances were recruited. Results: 477 Norwegian PWUD participated in the study. The mean age was 43.8 (SD 12.8) years and 77% were males. Thirty-four percent had injected drugs the past four weeks. Alcohol (41%) and cannabis (41%) were the most common drugs used the past four weeks, followed by tranquilizers (37%), central stimulants (35%) and opioids (30%). The majority (90%) had washed their hands frequently, used alcohol sanitizer during the past two weeks, had used face masks, kept one-meter distance to other people and stayed at home if feeling unwell. Fifty-four percent had been COVID-19 tested. More than half the sample (58%) had positive attitudes to COVID-19 vaccination, while 26% were fairly or very unlikely to accept vaccination. Those older (OR = 0.96, 95% CI 0.94; 0.98) and using face masks (OR = 0.49, 95% CI 0.30; 0.79) were more likely to have positive attitudes towards vaccination, while those reporting low life-satisfaction (OR = 3.86, 95% CI 1.43; 10.40), using opioids (OR = 2.97, 95% CI 1.43; 6.18) or almost never staying at home when feeling unwell (OR = 2.76, 95% 1.39; 5.45) expressed more negative attitudes towards vaccination. Conclusion: there was generally a high compliance towards COVID-19 recommendations, but one quarter of the sample was sceptical towards COVID-19 vaccination. This indicates a need for targeted and tailored information and well-designed vaccination roll-out programs to reach all PWUD.

## 1. Introduction

People who use drugs (PWUD) are assumed to be at particular risk of poor outcome of COVID-19 infection due to their use of substances and their way of living. They have an elevated risk of morbidity and premature mortality even without the COVID-19 pandemic [1,2,3]. During the pandemic it has been a growing concern regarding the risk of infection and its’ potentially devastating consequences within the PWUD community [4,5]. It is therefore important to protect PWUD from COVID-19 infection with preventive measures, such as social distancing and vaccination.

However, there are questions regarding compliance towards COVID-19 recommendations among PWUD. Less than optimal practices of social distancing among people with injecting drug use (PWID) have been reported [6]. It is possible that PWUD are less likely to practice social distancing as a consequence of having to buy substances illegally and of homelessness. It is important to know how the measures have affected the mental health of PWUD. One study found that depressive symptoms had increased among PWID during the pandemic [6]. Another study of people using opioids found that the feeling of isolation had increased during the pandemic [7]. The feelings of depression and isolation are likely to be a result of less social interaction. Consequently, there is a need for more studies on compliance to COVID-19 recommendations among PWUD and the consequences of these measures.

COVID-19 vaccination has proven to be important to prevent serious and fatal outcomes of a COVID-19 infection [8,9]. However, negative attitudes towards vaccination may work as major barriers against reaching all those at higher risk of a fatal outcome. In a general population study from United Kingdom, predictors of vaccine hesitancy were low-income, poor adherence to COVID-19 recommendations and female gender [10]. One of the main reasons for being unwilling to take the COVOD-19 vaccine were uncertainty around vaccine safety [11].

There are few studies examining PWUD’s attitudes towards COVID-19 vaccination. An Australian study found that only 57% would definitely or probably be COVID-19 vaccinated, while 15% reported that they would “definitely not” be vaccinated [12]. If the high proportion of vaccine hesitancy is representative for PWUD generally, this is of concern. It has been suggested that PWUD should be prioritized to vaccination and that roll-out plans should take account for specific barriers to uptake in this population [13].

The Norwegian government has monitored the COVID-19 situation closely and has issued recommendations according to the epidemiological situation in the country at different stages of the pandemic. Until December 2020 there had only been sporadic cases of COVID-19 infection among PWUD in Norway. However, in December 2020 there was an outbreak among PWUD in the city of Trondheim and later there was a large outbreak among PWUD in the city of Kristiansand as well as smaller outbreaks in other cities. In Kristiansand 63 COVID-19 cases, estimated to be more than 10% of the PWUD in the city, were confirmed [14].

In our 2020 survey from three major Norwegian cities during the initial phase of the pandemic [15], two thirds of the 226 PWUD were familiar with common COVID-19 symptoms, and 91% reported that they would take a COVID-19 test if experiencing relevant symptoms. The purpose of that study was further to find out if the respondents knew of the services available to COVID-positive PWUD and to examine drug availability and prices. The present study is a follow-up of our 2020 study focusing on other understudied issues.

To address these issues, we examined how a sample of PWUD complied towards the recommendations on how to avoid COVID-19 infection. We also examined the proportion having been tested for COVID-19 and their test results, social consequences of the pandemic and attitudes towards COVID-19 vaccination. Finally, we explored the associations between attitudes towards COVID-19 vaccination and relevant characteristics and behaviours. These findings may have important public health implications both in terms of the COVID-19 pandemic, but the findings may also be relevant in future pandemics where PWUD are at particular risks.

## 2. Materials and Methods

Design: Our cross-sectional study included 477 PWUD interviewed from 6 January to 15 March 2021 in 29 Norwegian cities/locations. Recruitment took place during the second/third wave of the pandemic in Europe and during the initial phase of the population-based vaccination programme. Any person above 16 years of age who used substances were recruited; there were no restrictions on type of substances used.

Recruitment of participants: We recruited people to recruit participants and perform the interviews through the users’ organizations and through different low-threshold services for substance users. As most participants were actively using drugs and did not have access to computers and/or patience to engage in an interview on their cell phones, we chose to recruit former drug users and professionals in low-threshold services to perform the interviews. An instruction was sent out to all the people conducting the interviews to ensure the interviews were performed in the same way. The interviews were performed by employees in low-threshold daytime service (24%) and detoxification units (16%), former drug users (13%), outreach street workers (13%), street clinic workers (11%), social service workers (8%), employees from low-threshold housing (7%) and treatment units (7%). Most interviews were conducted face-to-face, while 18% were performed on phone. We do not have information about the refusal rate. We had a close follow-up of the numbers of respondents during the period of recruitment, and stopped the survey when the numbers of new interviews became low. We categorized the cities/locations into the following geographical areas: “South East” (60% of the interviews), “West” (33%), “Middle” (7%) and “North” (9%) Norway. The corresponding population percentages for the geographical areas according to Statistics Norway from 2021 are 56%, 33%, 14% and 9%, respectively. 61% of the participants were recruited from “larger” cities with more than 100,000 inhabitants, while 39% of the participants were recruited from “smaller” cities/locations.

Setting: Norway has a population of 5.3 million inhabitants. Until March 2021 there had been 646 COVID-19 related deaths and 81,497 confirmed COVID-19 cases [16]. These figures were lower than in most other European countries. By 14 March 2021, 448,252 people had received their first vaccination dose [16]. In Norway, all inhabitants above 18 years were offered the COVID-19 vaccine, free of charge. Older age-groups and specific at-risk populations (not PWUD) were prioritized in the roll-out of the vaccination program.

Measures: We developed an interview-administered questionnaire with 29 items, including the background characteristics gender, age, current living situation and OMT status. The substance use behaviour items in the questionnaire were: “For the past four weeks”: “Have you injected substances”, “main drug of choice” and “additional substances used”. It also comprised a life satisfaction item: “How satisfied are you with life at the moment?”.

For COVID-19 preventive measures the following items were included: “In the past four weeks I have frequently washed hands”, “… used alcohol sanitizer when water has not been available” and “… used face mask when travelling on public transport and in shops”,” … kept at least one-meter distance to other people”, and “… avoided handshakes and hugging”. We also included the item: “I have stayed at home if feeling unwell”. The questions about washing hands, using alcohol sanitizer and using face masks, were yes/no type questions. The last three questions were answered on a five-point scale as shown in Table 2 in the result section. The COVID-19 preventive measure questions were aligned with the recommendations from the Norwegian Institute of Public Health at the time of the survey [17].

In addition, we included the following items for social consequences of the pandemic: “I have to a large degree isolated at home”, “I have less contact with my family”, “I have less contact with friends” and “I have felt more lonely”. The questions for social consequences were yes/no type questions.

We asked if the respondents had been tested for COVID-19 and their test results. Finally, we asked if the participants would be COVID-19 vaccinated, and if they were negative towards vaccination, we asked an open-ended question why.

We used compliance towards COVID-19 preventive measures, COVID-19 testing and test results, social consequences of the pandemic and the participants’ intention to be COVID-19 vaccinated as key variables to describe the sample.

The questionnaire was tested out on approximately five patients before it was implemented in the survey. The interviews were documented directly on an I-pad/computer by the employees/former drug users, with the answers being uploaded directly into the database at the Services for sensitive data (TSD) at the University of Oslo. The other option was to use a paper and pencil questionnaire, thereafter the first author (GWS) entered the answers into the database. The questionnaire took five to ten minutes to complete.

The following procedures were followed in order to avoid participants answering to the questionnaire more than once: Firstly, there was a question at the beginning of the survey asking if the respondent had answered the survey previously. Secondly, when “washing the data-set”, we removed some respondents who most probably had answered the survey twice.

In Tables 2 and 3 (see result section) there are some missing data, as not all the responders answered to every question. The missing data were present both in the paper questionnaires and in the online questionnaires. We decided to include incomplete data sets and account for them because: (1) The responders are active drug users and often have a shorter attention span. (2) Data sets where many questions were unanswered were excluded. We decided it was valuable to include the answers from the responders where most of the questions were answered.

Analyses: We conducted the statistical analyses in Stata 16.0. Multivariable logistic regression analysis was used to examine the associations between attitudes towards vaccination and demographics, life satisfaction, and compliance with COVID-19 preventive measures. The outcome in the logistic regression analysis was being negative towards COVID-19 vaccination. Unadjusted odds ratios (OR) and adjusted odds ratios (aOR) with 95% Confidence Intervals (CI) were reported.

The responses to the question “How likely are you to be COVID-19 vaccinated if the vaccine is recommended to you?” measured the attitudes to vaccination. The item was answered with the responses “very likely”, “most likely” “neither or/neutral”, “most unlikely”, and “very unlikely”. We aggregated the attitudinal item into a dichotomous item labelling the categories “positive or neutral” or “negative”. The first category comprised those responding they were very or most likely to be vaccinated or neutral. The negative category comprised of those stating they were very or most unlikely to be vaccinated.

Previous studies have found gender, age and socioeconomic status to be associated with attitudes towards COVID-19 vaccination [17,18], and we therefore included these as dependent variables into the logistic regression analysis. We included homelessness/shelter use as a proxy for lower socioeconomic status. Furthermore, a high score on a depression and anxiety checklist (the Hopkins Symptom Checklist (HSCL-5)), have been associated with being negative to the COVID-19 vaccine [17]. We did not have a depression score in our survey, but we hypothesized that there might be an association between attitudes towards COVID-19 vaccination and life satisfaction. We are not aware of any previous studies examining the associations between attitudes towards COVID-19 vaccinations and substance use behaviours, nor compliance to COVID-19 preventive measures. However, we included these variables for exploratory purposes.

The dependent variable in the multivariable logistic regression model was being unlikely to accept COVID-19 vaccination. The reference category was being likely or neutral to COVID-19 vaccination. The independent variables included into the model were male gender, age, homelessness/shelter use, life satisfaction, injection past four weeks, main drug of choice past four weeks, frequent handwash, used “antibac” when water was not available the past two weeks, used mouth masks on public transport and in shops past two weeks, “Do you stay at home if feeling unwell?” and recruitment area. Only three responses were missing from the dependent variable and another 25 responses were missing from the independent variables. Due to the small number of missing values of these covariates ranging from three for the variable “homeless/shelter user” to 10 for the variable “using face masks” the analytic n was reduced to 449 in the regression model through the default listwise deletion. No advanced approaches were used because of this low n.

Ethics: This study is a follow-up of a previous study conducted May–June 2020. Prior to the data collection the study protocol was submitted to the Regional Ethics’ Committee and the Data Inspectorate at the University of Oslo. Both bodies concluded that the questionnaire was anonymous and therefore did not need approval. As the current study is a follow-up of the previous study and no additional personal identifying information were included into the questionnaire an approval was deemed unnecessary.

We gave a short sheet of information to each participant. In the questionnaire, the respondent had to confirm that he/she had received the information.

## 3. Results

The majority (77%) of the 477 participants were males, and the mean age was 43.8 years, range 16 to 79 years (Table 1). Approximately 1/5 of the sample were homeless or shelter users, and 36% were currently in OMT. Alcohol (41%) and cannabis (41%) were the most reported substances used, followed by tranquilizers (37%), central stimulants (35%) and opioids (30%).

Ninety-four per cent of the participants had washed their hands frequently during the past two weeks and used alcohol sanitizer when water was unavailable (Table 2). Eighty per cent of the sample had also used face masks when travelling on public transport and visiting shops and had always or almost always kept at least one-meter distance to other people and avoided shaking hands and hugging. Additionally, eighty-three per cent had stayed at home if they felt unwell during the pandemic.

**Table 2:** Compliance towards COVID-19 preventive measures, testing and test results and social consequences of the pandemic in a sample of 477 PWUD.

	Total (*n* = 477)
How do you think the follow-up you receive during the pandemic has been?	
Better than pre-COVID-19	20% (91)
Same as pre-COVID-19	55% (256)
Poorer than pre-COVID-19	26% (120)
*In the past two weeks I have conducted*	
Frequent handwash	94% (443)
Used alcohol sanitizer when water not available	95% (448)
Used face mask on public transport and in shops	80% (375)
Kept at least one-meter distance to other people	
Yes, always or almost always	83% (391)
Yes, sometimes	12% (58)
Almost never	4% (20)
No, not applicable	1% (5)
Avoided handshakes and hugging	
Yes, always or almost always	84% (397)
Yes, sometimes	10% (48)
Almost never	5% (23)
No, not applicable	2% (7)
During the pandemic, I have stayed at home if feeling unwell (also when mild symptoms)	
Yes, always or almost always	83% (392)
Yes, sometimes	6% (27)
Almost never	4% (18)
No, not applicable	8% (38)
Have you been tested for COVID-19?	
Yes, once	26% (125)
Yes, several times	29% (138)
No	45% (213)
If yes, what was the test results?	
Negative	96% (232)
Positive	2.5% (6)
Awaiting test results	1.7% (4)
During the pandemic I have	
to a large degree isolated at home	62% (288)
less contact with my family	49% (224)
less contact with friends	63% (293)
felt more lonely	51% (236)

Missing: 6 population at risk, 10 follow-up during the pandemic, 1 received sufficient information. 3 suspect current or previous COVID-19, 5 hand wash, 7 alcohol sanitiser, 7 face masks, 3 one-meter distance, 2 handshakes and hugging, 2 stayed at home if unwell, 12 isolated at home, 18 less contact with family, 14 less contact with friends, 18 more lonely.

More than half the participants had been tested for COVID-19 at least once, and among those tested, 2.5% reported that they had a positive test result (Table 2). The most commonly reported social consequences of the pandemic was to have less contact with friends (63%) and to a large degree have been isolated at home (62%) (Table 2).

Slightly more than half the participants stated that they were very (43%) or fairly (15%) likely to be COVID-19 vaccinated, while 16% stated that they were neutral. A quarter of the respondents reported that they were fairly (8%) or very (18%) unlikely to be vaccinated. Overall, 99 participants responded to the open-ended question regarding why they were negative to vaccination (Table 3). Of these, 31% stated that they did not trust the COVID-19 vaccine or those producing it, while 27% stated that they were afraid of short- and/or long-term side-effects.

**Table 3:** Reasons for being negative to COVID-19 vaccination in a sample of PWUD (*n* = 99).

	Total 100% (*n* = 99)
Do not trust the vaccine	31% (31)
Afraid of side-effects	27% (27)
Afraid of becoming ill and die from the vaccine	10% (10)
There is not enough research	11% (11)
Critical towards vaccine(s)	7% (7)
Not afraid of COVID-19	5% (5)
Do not believe in COVID-19	2% (2)
Other reasons	6% (6)

Low life-satisfaction, opioid use, and almost never staying at home if feeling unwell increased the likelihood of a negative attitude towards vaccination, while increasing age and using face masks in public decreased the likelihood (Table 4).

**Table 4:** Predictors for being negative towards COVID-19 vaccination in a sample of PUD (*n* = 452) estimated using univariate and multivariable logistic regression analysis. Being positive or neutral towards COVID-19 vaccination comprise the reference category.

	Positive or Neutral *n* = 350	Negative *n* = 124	OR (95% CI)	aOR (95% CI)
Male	78.% (273)	74.0% (91)	0.80 [0.50, 1.29]	0.84 [0.48, 1.46]
Mean age (SD)	45.4 (12.8)	39.2 (12.0)	0.96 [0.94, 0.98] ***	0.95 [0.93, 0.97] ***
Homeless/shelter use	16.6% (58)	27.4% (34)	1.90 [1.17, 3.09] *	1.43 [0.79, 2.61]
Overall, how satisfied are you with life at the moment?				
very satisfied	10.0% (35)	6.6% (8)	1.00	1.00
Fairly satisfied	35.5% (124)	28.9% (35)	1.23 [0.53, 2.90]	0.95 [0.38, 2.37]
Neither or/neutral	30.% (106)	36.4% (44)	1.82 [0.78, 4.23]	1.51 [0.60, 3.78]
Fairly dissatisfied	18.1% (63)	13.2% (16)	1.11 [0.43, 2.86]	0.69 [0.24, 1.98]
Very dissatisfied	6.0% (21)	14.9% (18)	3.75 [1.39, 10.12] **	3.25 [1.09, 9.70] *
Injected past four weeks	30.7% (107)	43.1% (53)	1.71 [1.12, 2.61] *	0.82 [0.44, 1.51]
Main drug of choice past four weeks				
Alcohol	22.9% (80)	13.8% (17)	1.00	1.00
Opioids	10.0% (35)	19.5% (24)	3.23 [1.54, 6.75] **	3.27 [1.28, 8.40] *
Central stimulants	14.0% (49)	21.1% (26)	2.50 [1.23, 5.06] *	2.02 [0.81, 5.01]
Other substances	37.4% (131)	35.0% (43)	1.54 [0.83, 2.89]	1.47 [0.70, 3.10]
Not used any substances	15.7% (55)	10.6% (13)	1.11 [0.50, 2.47]	1.18 [0.47, 2.95]
Frequent handwash past two weeks (ref = no)	96.0% (333)	87.7% (107)	0.30 [0.14, 0.64] **	0.51 [0.18, 1.39]
Used alcohol sanitizer when water not available past two weeks (ref = no)	97.1% (336)	90.1% (109)	0.27 [0.11, 0.64] **	0.48 [0.13, 1.77]
Used face mask on public transport and in shops past two weeks (ref = no)	82.9% (286)	70.5% (86)	0.49 [0.31, 0.80] **	0.49 [0.27, 0.89] *
Do you stay at home if feeling unwell (also when mild symptoms)?				
Yes, always	57.1% (201)	49.6% (61)	1.00	1.00
Almost always	29.0% (102)	22.8% (28)	0.90 [0.54, 1.50]	0.66 [0.37, 1.17]
Yes, sometimes	6.0% (21)	4.9% (6)	0.93 [0.36, 2.41]	0.65 [0.22, 1.90]
Almost never	2.0% (7)	8.9% (11)	5.12 [1.90, 13.80] ***	3.96 [1.22, 12.89] *
No, not applicable	6.0% (21)	13.8% (17)	2.64 [1.31, 5.32] **	1.41 [0.56, 3.54]

Three missing from the outcome variable “view on COVID-19 vaccination”. From the predictor variables the following were missing: 4 gender, 11 age, 3 homeless, 7 life satisfaction, 5 injecting, 5 main drug of choice, 8 handwash, 10 alcohol sanitizer, 10 face masks, 5 stay at home. * =< 0.05, ** =< 0.01, *** =< 0.001.

## 4. Discussion

There was high compliance towards COVID-19 recommendations among PWUD about one year into the pandemic. The majority of participants had recently washed their hands frequently, worn face masks in public, kept social distance and stayed at home when feeling unwell. Approximately a quarter of the sample had negative attitudes towards COVID-19 vaccination.

The proportion of the sample reporting to comply to social distancing recommendations was similar to the findings in a US study among OMT patients attending a methadone clinic, however a higher proportion of our respondents reported to stay at home when feeling unwell [19]. The high compliance to the recommendations was contrary to the anecdotal stories from people working in the field where compliance had been reported to be low. However, information about the refusal rate was not available, the finding might therefore be partly attributable to selection bias.

The most common reported social consequence of the pandemic was to have less contact with friends and to have isolated at home. It is therefore not surprising that half the sample reported that they had felt lonelier during the pandemic. This is in line with an OMT study where 47% reported increased loneliness [19]. One quarter of the sample reported that they were currently dissatisfied with life. This is in line with a general population study from Norway from late 2020 which found that 24% were dissatisfied with life [20].

The proportion of participants in our study reporting positive COVID-19 test results (2.5%) was on the same level as reported in a Norwegian general population study where 1.9% of the tests were positive [16].

Vaccines are assumed to be the primary intervention to combat the COVID-19 pandemic. Slightly more than half the participants reported that they were positive towards COVID-19 vaccination. This is much lower than what has been found in general population surveys conducted in Norway, where 73% stated that they were positive towards COVID-19 vaccination [17,21]. Yet this is in line with a study of PWID where 57% stated they would take the COVID-19 vaccine [12].

Approximately one quarter of the sample was negative towards COVID-19 vaccination. This is in line with an Australian study, where 15% of PWID in December 2020 reported that they would definitely not take the COVID-19 vaccine and 7% reported they would probably not take the vaccine [22]. However, this is higher than what has been found in other surveys conducted in Norway in late 2020 and early 2021 [17]. However, the proportions being negative towards vaccination in our study were more in line with people with immigrant background, where 20–30% reported being negative towards vaccination [17]. It might seem that those with immigrant background and PWUD, both often recognized as marginalized groups, are more sceptical towards COVID-19 vaccination than the general population.

In line with previous Norwegian studies from the general population, older age was associated with being less negative towards vaccination [17]. However, those very dissatisfied with life, and almost never staying at home if they felt unwell were more likely to be negative towards vaccination compared to those very satisfied with life and those always or almost always staying at home. The combination of being skeptical towards vaccines and the lack of adherence to COVID-19 recommendations such as social distancing is particularly troublesome and risky. The most common reason for being negative towards vaccination was lack of trust in the vaccine and fear of side-effects. In an Australian study of COVID-19 vaccination, safety concerns were the most common reasons for not wanting a COVID-19 vaccine [22].

Our findings show a mixed pattern: The majority of our respondents seem to comply with the recommendations on how to avoid COVID-19 infection. On the other hand, some PWUD seem to be more sceptical to COVID-19 vaccination than the general public [16,17]. This emphasizes the need for targeted and tailored information and well-designed vaccine roll-out programs to PWUD and other marginalized populations. A particular challenge is therefore to design promotional information regarding COVID-19 vaccination that will reach persons experiencing social isolation and low life satisfaction.

### Strengths and Limitations

This study gives a unique insight into how the pandemic and its responses were perceived in a sample of PWUD in Norway one year into the COVID-19 pandemic.

The strength of the study is that it is performed during the COVID-19 pandemic with emphasis on questions relevant for the government, PWUD, clinicians and low-threshold workers, in order to streamline the information and recommendations necessary to combat the pandemic among PWUD.

On the other hand, there are some study limitations. Self-reported interview data are open to recall bias, under- and over-reporting followed by imprecise estimation. They are also open to social desirability bias. Further, we find it unlikely that participation in the study was perceived as a way to enhance access to care and services for the responders. Low-threshold services in Norway provide care and follow-up to all PWUD using the services. Interviewers also reported that the survey was a gate-opener to discussions about COVID-19.

Our sample is also relatively small, so our results have to be interpreted with caution. Further, there are missing responses to some of the questions in the survey. Intention to vaccinate is not the same as actually taking the COVID-19 vaccine. We have however, compared the intention to vaccinate in our sample with intention to vaccinate in other studies. Although, the findings from our regression analysis are in line with previous studies examining vaccine hesitancy, the results must be interpreted with caution due to the small study sample resulting in wide confidence intervals in some of the estimates in the model. Three participants had not responded to the outcome variable “view on COVID-19 vaccine” and another 25 participants had incomplete responses in one or more of the predictor variables in the regression model in Table 4. These results therefor need to be interpreted with caution.

Future research should focus on actual vaccination rates among PWUD and find out how many doses of COVID-19 vaccines PWUD have taken compared to the general population. This will be important results in order to plan for future vaccinations for COVID-19 and other viruses among PWUD.

## 5. Conclusions

The participants reported to comply with the recommendations on how to avoid COVID-19 infection. On the other hand, ¼ of the sample was sceptical towards COVID-19 vaccination. There is a clear need for targeted and tailored information and well-designed vaccination roll-out programs for PWUD. In order to achieve this, decision-makers need to identify specific challenges in terms of marginalized sub-populations and their needs.

## Figures and Tables

**Table 1:** Baseline characteristics in a sample of 477 PUD recruited January to March 2021.

	Total
	*n* = 477
Male	77% (364)
Mean age, years (SD)	43.8 (12.8)
Homeless/shelter user	20% (93)
In OMT	36% (172)
Injected drugs past four weeks	34% (162)
Total number of substances used, mean (SD)	2.3 (1.2)
Substances used past four weeks *	
Alcohol	41% (193)
Cannabis	41% (195)
Tranquilizers	37% (176)
Central stimulants	35% (167)
Opioids **	30% (141)
Other	10% (46)
Not used any substances	14% (66)
Overall, how satisfied are you with life at the moment?	
Very satisfied	9% (44)
Fairly satisfied	34% (159)
Neither or/neutral	32% (150)
Fairly dissatisfied	17% (81)
Very dissatisfied	8% (39)

Missing: 1 male, 8 age, 2 injected, 4 main drug of choice, 2 OMT, 4 life satisfaction. * The categories are not mutually exclusive, ** Excluding own OMT medication.
